# Discipline-Based Education Research for Animal Welfare Science

**DOI:** 10.3389/fvets.2020.00007

**Published:** 2020-01-28

**Authors:** Jill R. D. MacKay

**Affiliations:** Royal (Dick) School of Veterinary Studies, The University of Edinburgh, Edinburgh, Scotland

**Keywords:** education, animal welfare, education research, interdisciplinary research, scholarship of learning and teaching

## Abstract

Animal welfare science features interdisciplinary and collaborative working across fields, spanning behavioural ecology, psychology, veterinary sciences, economics, and fundamental biology. However, education research is not yet prevalent within the animal welfare literature. In a Web of Science topic search there were 188 papers which specifically discussed or explored how to teach animal welfare from 1978 to 2017. Of these, only 34% (*n* = 61) specifically focused on instructional design or pedagogical research, and these were predominantly within veterinary education (57%). Despite this, the literature is in broad agreement that animal welfare education is an important topic that should be done well. Within the UK, there were a possible 586 animal-related courses within Universities College Admissions Service database for potential students to choose from, highlighting the significance of robust and considered educational practice. The current gaps identified in the literature were discussion of hidden curriculums outside of veterinary degrees, animal-centered education, the blueprinting of assessment, and authentic assessment. Therefore, this review proposes that animal welfare scientists interested in education consider discipline based educational research (DBER) practices, and engage more fully with the educational research literature. A key component of DBER is the recognition that specialist knowledge needs to be taught by specialists, and so it is important that animal welfare scientists begin to access educational research too.

## Introduction

In the UK, the Joint Academic Coding System (JACS) is used by the Universities College Admissions Service (UCAS) to classify academic subjects (1). These codes broadly categorise what an undergraduate degree programme within the UK will teach. There are five JACS codes we might expect to relate to animal welfare teaching, C300 (Zoology), D100 (Pre-clinical veterinary medicine), D200 (Clinical veterinary medicine and dentistry), D300 (Animal Science), D400 (Agriculture), and D900 (Other subjects in veterinary science, agriculture and related subjects). As of late 2017, the UCAS website (1) reported there were a possible 586 animal-related courses available to students entering further or higher education in the UK. These options may be reasonably expected to grow, with recent proposals for a ninth veterinary school between Keele University and Harper Adams (2), and an expansion of agricultural programmes at the University of Edinburgh (3). As the UK moves toward an uncertain legislative front with questions around how the veterinary profession and animal research will withstand the UK's planned departure from the EU, animal welfare concerns are increasingly reported within the media (4–6). While these concerns are uniquely pressing for the UK, we have also seen growing evidence of animal welfare concern internationally, with China implementing animal welfare legislation (7), and growing need for veterinary and agricultural teaching on an international level (8, 9). It is clear that we are approaching a critical period for animal welfare science. As we look toward global food security and safety challenges (10), it is of fundamental importance that animal welfare is taught as a scientific discipline, and is incorporated within all relevant courses. The UK Higher Education Statistics Agency (HESA) estimates that between 48 and 99% of graduates from the aforementioned courses will go on to professional occupations (11), where they may well make decisions affecting animal welfare at all levels of society. Do we give animal welfare teaching the attention it deserves?

It has been argued that animal welfare science is innately interdisciplinary (12, 13), requiring an understanding of behavioural ecology, psychology, veterinary sciences, economics, fundamental biology, ethics, anthropology, and the ability to communicate between these different fields. As this paper will demonstrate, this interdisciplinary focus has not always included education research. Indeed, much of the specific animal welfare education research has been focussed around determining that a need for animal welfare education exists [as in (14–16)]. Given the current socio-political environment, and the rising importance of animal welfare, this paper aims to explore the current understanding of animal welfare education in the literature, and lay out recommendations for future work.

### The Place of Animal Welfare Scientists in Discipline Based Education Research

Before examining the literature, it is worth questioning whether animal welfare scientists are best placed to explore education in animal welfare. At the International Society for the Scholarship of Teaching and Learning 2017, a panel discussion explored the crossover between Scholarship of Teaching and Learning (SoTL), Educational Research as a discipline within itself, and Discipline Based Educational Research (DBER), and the conflicts that can arise between these fields (17). Educational research is a broad term, concerned principally with the methodology used to explore learning (18). Perhaps it is not a coincidence that Nisbet highlights the efforts of animal behaviour scientists such as Edward Lee Thorndike in founding educational research in Europe. Educational research therefore has strong links with psychology and child development. Mortimore (19) describes the aims of educational research as (1) to systematically observe and record, (2) to analyse and draw out implications, (3) to publish findings, and (4) to attempt to improve educational attainment. However, Mortimore also highlighted that educational research often featured poor quality work and rightly faced criticism regarding its occasional biases. SoTL has emerged as a term which slightly competes with educational research, perhaps with a more applied focus. Miller-Young and Yeo (20) stated that SoTL's goals were to deepen an educator's understanding of student learning and explore the effectiveness and desirability of what we do in higher education. SoTL is often targeted by educational researchers as a “weaker” version of what education research is itself (21), however SoTL bears with it an underlying assumption that any expert who teaches a subject must seek out enough pedagogical understanding in order to effectively maintain the production of future experts (22). SoTL is therefore more firmly linked to higher education or specialist education where it is understood that a generalist, who may understand the principles of learning very well, may not understand the intricacies of a particular subject. This leads us to DBER. The goals of DBER within science have been stated as (1) understand how people learn concepts, practices and ways of thinking of science and engineering, (2) understand the nature and development of expertise within a discipline, (3) identify and measure appropriate learning objectives and instructional approaches, (4) contribute to the knowledge base in a way that can guide the translation of DBER findings to the classroom, and (5) identify ways we can make science and engineering more inclusive (23). Although keen readers will have spotted that DBER's aims, which come from work explored by the National Research Council, do not follow principles of good instructional design themselves [as we know, “understanding” cannot be assessed (24)]. There is clearly great overlap between these three areas, but the subject-specific import of SoTL and DBER approaches hold particular interest. They highlight that within-discipline knowledge exchange, be that from researcher to undergraduate, or researcher to the public, is an essential component of modern research (25).

### Epistemological Assumptions of This Review

Within education research there is also the issue of epistemology, which will be familiar to animal welfare researchers with more grounding in the philosophies. An epistemology is a theory of knowledge, and can be simplified as “what does it mean to ‘know’ a fact?” Within research a positivist epistemology is encouraged, sometimes implicitly, by the dominance of Science Technology Engineering Mathematics and Medicine (STEMM) fields and the reward structures of research (26). Positivism is often framed in opposition to constructivism (27), in that positivism views facts as objective truths which can be uncovered whereas constructivism views the truth as a socially negotiated entity. Where scientific writing is ignorant of epistemology, it often ultimately favours positivist leanings (26), as scientists write in a highly ritualised manner with highly empiricist and positivist viewpoints (28). As constructivist writing in science has been criticised as being full of “jargon” (29), I will state plainly that in this paper I write from my individual viewpoint. I present my experience where I believe it is useful to contextualize the opinions I put forth, so that the reader may critically appraise the material. This is sometimes unfamiliar to many STEMM readers but in a discussion of DBER it is important to include reflective practice as an aspect of education research (30). The aim of this paper is to explore how a DBER approach to animal welfare education can support the animal welfare field through providing a repeatable literature review and a critical evaluation of the educational literature aimed at animal welfare scientists.

## Materials and Methods

First, I performed a literature review to characterise the animal welfare education field, and then synthesised the results with my own knowledge of the education research literature to critically evaluate any gaps where DBER can support animal welfare education. The literature review was performed in a repeatable manner, to allow for other work to build on this methodology, and afford the opportunity to re-evaluate how any DBER approaches may influence the field in future.

### Conducting the Literature Review

The literature review was conducted using Web of Science's search tools, as this provides meta-data [see (31)] such as citation count and keywords which can provide some indication of an article's reach and impact. While Web of Science is not an exhaustive database, it is the most conservative in terms of this meta-data compared to Google Scholar and Scopus (32) in that it is less likely to view a non-peer reviewed webpage citation as a “true” citation. Due to the popularity of animal welfare discussions on the internet, I considered this a benefit of using only Web of Science searches for this review.

In October 2017, I conducted a Web of Science search concerning “animal welfare” and “education” as key words using BOOLEAN search terms, i.e., the search would only return articles which reference both topics. This returned 406 articles published over a 39 year period. This search was exported as a text file using Web of Science's search tools for further refinement. I reviewed the text of these publications and 200 were excluded for not specifically discussing animal welfare in an educational context, e.g., they proposed further education would improve animal welfare, or explored the impact of education on attitudes to animal welfare. This excluded a large number of studies about consumer choice behaviour with regards to animal products. Studies were retained where they explored students' perceptions of animal welfare, as participants in these studies were being recruited specifically because of their student role. A further three studies were excluded for being duplicate records. Excluding book reviews, news items, and editorial materials, there were 188 publications from 1978 to 2017 relating to education and animal welfare.

All but 10 papers were available to the author for collection, or possessed abstract information in Web of Science's database. After reviewing the papers they were assigned to broad categories to characterise the type of animal considered in the study, the educational audience, and the “purpose” of the paper. These categories are defined in Table 1. X^2^ analyses were used to explore differences in observed numbers of papers across the categories in R Version 3.4.2 (Short Summer) from the R Foundation for Statistical Computing and R Studio. The “textstem” package was used to lemmatise the abstracts of these articles. Lemmatisation is a form of language processing which stems words with reference to their grammatical origin, e.g., “running,” “runs,” and “ran” would be shortened to “run,” while “runner” would be retained as independent to “run”. Then the tidytext package (33) was used to strip data from the abstracts of these papers and explore most frequent words through the use of document term matrices. All data processing code is available in the Supplementary Materials.

**Table 1:** Category definitions for 178 papers regarding animal welfare education.

**Category**	**% Paper (N)**	**Definition**
**Animals**		
General	48.9% (87)	Intended to apply to all animals
Companion animals	10.7% (19)	Animals kept in a companion role, commonly dogs, cats, rabbits, etc.
Equine	1.7% (3)	Equids, can include working and companion
Production animals	18.0% (32)	Animals in farm or production settings
Research animals	16.3% (29)	Animals kept for the purposes of research, mainly within laboratory environments
Captive free ranging animals	3.9% (7)	Free-ranging animals kept under direct human management for the purposes of entertainment, conservation or education
Free ranging animals	0.6% (1)	Free-ranging animals living outwith direct human management
**Audience**		
Adolescents	1.7% (3)	Teenagers outside of an academic context
Children	7.3% (13)	Young children (<13), may be inside schooling context
Industry	19.1% (34)	Researchers, agricultural industry, zoological industry
General higher education	10.1% (18)	Students studying at a higher education level
Public	11.3% (20)	Public engagement or consumer roles
Educators	0.6% (1)	
Veterinary	50% (89)	Both veterinary students
**Purpose**		
Attitudinal study	22.5% (40)	Exploring the effects of education on attitudes
Call to action	19.1% (34)	Consolidates evidence from a variety of sources to provide informed opinion about future practice
Practice review	23.6% (42)	Consolidates evidence from a variety of sources to provide and informed opinion about current practice
Pedagogical study	34.3% (61)	Explores some aspect of instructional design in animal welfare topics
Animal welfare in education	0.6% (1)	Explores the welfare of animals within an education setting

## Results

### Current Views on Animal Welfare in Teaching

**Figure 1:**
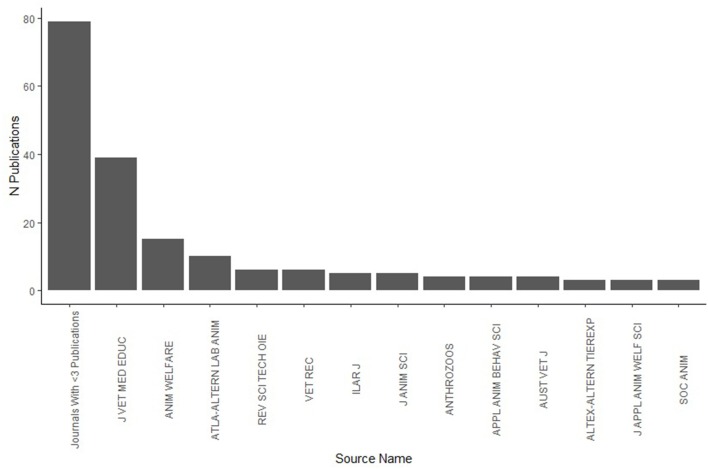
Publications (*n* = 188) by source title in Web of Science Search. Topic Boolean search string: “Animal Welfare” and “Education”.

**Figure 2:**
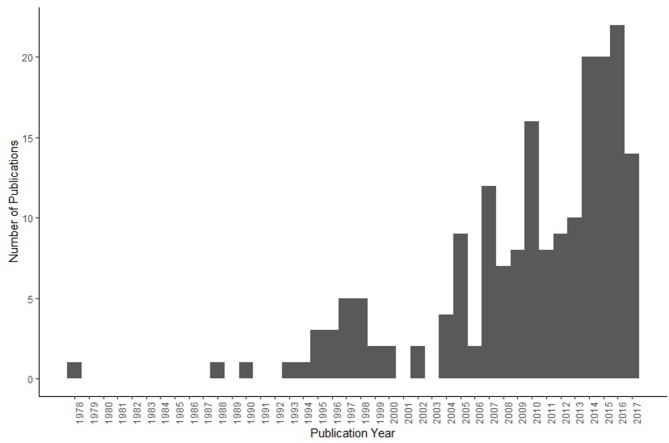
Publications by year (*n* = 188) in Web of Science Search. Topic Boolean search string: “Animal Welfare” and “Education”.

The majority of papers were published in the Journal of Veterinary Medical Education (*n* = 39, 21%), with Animal Welfare (*n* = 15, 8%), and ATLA-Alternatives to Laboratory Animals (*n* = 10, 5%) representing the three most populous journals. Overall, 79 papers (42%) were published in journals with fewer than three papers on animal welfare education (Figure 1). In addition, the majority (71%) had been published since 2008 (Figure 2).

**Figure 3:**
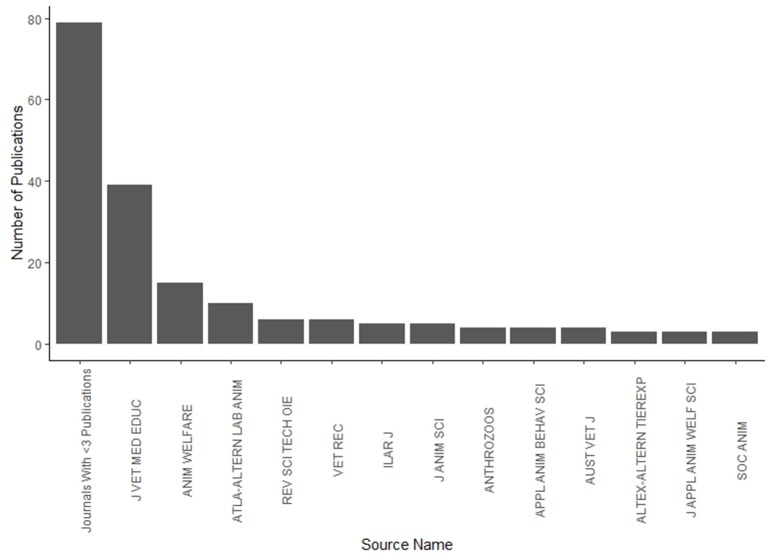
Frequency of lemmatised words present in 188 abstracts regarding animal welfare education.

The distribution of paper “purpose” was non-random across the type of animal in a Chi^2^, but perhaps this is to be expected. However, there were significantly more “practice reviews” on the topic of research animals [$\chi_{\left(35\right)}^{2}$ = 268.58, *P* < 0.001], and the odds of a paper about research animals being about a practice review were 9.2 times higher than other animal categories. The 20 most common words found in the abstracts are depicted in Figure 3.

### Summary of Current Animal Welfare Education Research

There is a broad consensus within the literature that animal welfare education is an essential part of many animal-related curricula, from primary school (34) to high school (35), the veterinary curriculum (36), and to people working within animal industry (37). Much of the focus of the papers published on animal welfare education focus on higher education (60% of the papers sampled, across veterinary, and general higher education subjects), and all papers supported the provision of animal welfare teaching at this level in animal or society related subjects.

Of pedagogic approaches, digital education is a common topic within the higher education sector (38–42) and industry environments (43–45). This may seem surprising initially, as digital education is often synonymous with “distance learning,” and distance learning presents a challenge for the teaching of practical skills (46). Conversely, animal welfare education is often spoken of in terms of skills, from clinical skills for vets to communication and management skills (47). Other work has shown that within farm animal welfare education across Europe, the interactivity of teaching is greatly variable (48), so it is wise not to assume that on-campus teaching is inherently more practical-focussed. Some of the studies around digital education and animal welfare have explored whether this results in a more theoretical and less applied understanding of animal welfare. For example, Klupiec (40) found that veterinary students often “missed” the practical application of work when working solely from e-learning resources about animal handling. However, the rise of digital education papers does not appear to be from a belief that digital spaces are particularly well-suited for teaching animal welfare, but because the demand is so high, and time often in short supply. Of the 20 papers aimed at educating the public, four explored digital education for the public and whether the provision of these resources could be used to improve animal welfare knowledge more generally (49–52). These papers highlighted that digital resources, when well-designed, can offer flexible learning opportunities at a pace that suits the student, and is suited for global education initiatives.

Despite this strand of producing open educational resources for public consumption, continuing education was most often discussed for veterinary or research professionals, with a number of papers exploring the poor flow of information between science and industry (53, 54). For example, Algers et al. (38) criticised higher education institutes for not making more open educational resources about this societally-important issue. However, there are challenges in developing resources for professional industries. Algers and Berg (55) conducted a case study on controversy surrounding slaughter and why this affected the creation of open educational resources. They highlighted that educators worried about discussions of the learning materials rapidly becoming “polarised”. Zuin et al. (56) performed a qualitative evaluation of a “dialogical” course in animal management in Brazil, and the participants of this study highlighted that students drawing their own opinions from the material presented was important. Although this study also highlighted some differences in practice between the animal handlers and the trainers. Regardless, the sensitivity of animal welfare topics present as a barrier to teaching in many studies, but may be a space for a different pedagogic approach (see patient-centred teaching below).

A large number (22%) of the papers on animal welfare education are attitudinal, e.g., does education have a positive or negative effect on attitudes, or do vets lose empathy (57) or find animal welfare discussions a challenging component of their job (58). Attitudinal research is undeniably important within human-animal interactions and animal welfare education research, but it has its limits. It is also important not to confuse either educational attainment nor attitudinal change with behavioural change. There is extensive evidence from the field of public health and environmental sustainability demonstrating that the link between knowledge, attitudes and behaviours is complex (59–61), and there is considerable discussion in the literature about behavioural change theories such as the Theory of Planned Behavioural Change (62, 63). Animal welfare education studies should be cautious of claiming that human behaviours will be changed because a short term change in attitudes or knowledge has been attained.

### Current Gaps in Animal Welfare Education Research

There are a number of areas of education research which would be pertinent for animal welfare educators to explore. Many of these have already been touched on within veterinary education, although not always with an understanding of how they might affect animal welfare teaching. There are four main areas which may be of particular interest to animal welfare educators: the role of hidden curriculums; the concept of patient-centred education; the use of instructional objectives or learning outcomes to blueprint assessment; and the role of authentic assessment in a practical subject. This is by no means an exhaustive list of topics which may be of interest to the animal welfare educator, but includes topics which should be familiar to those who research attitudes to animal welfare, and key areas where there is room for improvement in how we think about teaching animal welfare across a variety of levels.

#### Hidden Curriculum

Sambell and McDowell (64) referred to the “hidden curriculum” as the difference between what institutions intend to teach, and what learners experience “on the ground.” The seminal report on hidden curriculums comes from Snyder (65) who detailed its formation in the Massachusetts Institute of Technology, and presented to students principally a focus on achieving the right exam results over developing well-rounded degree experiences. Snyder (65) details examples such as the student who learns how to precisely follow protocol and take the minimum amount of risk in order to achieve their predicted grades, generating an unpleasant higher education experience. Of note to the animal welfare scientist, Snyder spends some time in chapter one defining “adaptive mechanisms,” “adaptation,” and “coping patterns” with respect to students.

“*Coping patterns I take to refer to some behaviour, some action which alters the individual's relationship to his environment. A coping pattern, in order to be so named, must have some influence on the individual's adaptation to the environment by altering his behaviour in relation to that environment*.” [(65), p. 11].

Upon my first reading of Snyder's book I was struck by the connection between education research and animal welfare science, as I would not consider this definition of coping patterns out of place in animal welfare literature.

The relevance of hidden curricula to animal welfare education goes beyond a commonality between student coping and animal coping. They are probably one of the more explored pedagogical theories in reference to animal welfare science. For example, they were discussed as part of the veterinary curriculum by Dolby (66), who emphasised that as veterinarians are a key point of contact for the public's understanding of animal welfare, hidden curriculums within veterinary teaching have a long-lasting impact. The formation of these hidden curriculums within veterinary teaching have been discussed as a reason for a lack of veterinary empathy by Degeling et al. (67), and it is recognised that much of this occurs in the workplace as well as within the classroom (68). Hidden curriculums were referenced in a veterinary animal welfare teaching context, though not explicitly discussed by Whittaker (16), Batchelor et al. (69), and Dolby and Litster (70).

The hidden curriculum impacts both student and animal. I would claim that the majority of students who go in to animal-related subjects do so because they have an innate affection for animals. They want to work with or help animals. Incidentally, while there is some research exploring why potential veterinarians choose their degrees (71, 72), there is less work exploring this in other animal-related professions although the “sense of calling” seems to be important here too (73). In animal welfare education we must guard against a passive message that grades are more important than this initial passion for the subject. This does not imply that we lower standards, but that we encourage well-rounded practitioners of animal welfare who are self-reflective and critical, and not dependent upon a simple view of education that there is one right answer. As Snyder highlights, simplistic right/wrong views of education can produce frustrated students who are unable to create a conversation about what they want out of their educational experience. If our hidden curriculum fosters a fundamental conflict in a person's core values, it will inevitably produce unhappy students.

Hidden curriculums are thought to arise mainly from the socialisation of the student into the unwritten culture of the teaching department (74). In this way, I personally find constructivism a useful lens through which to conceptualise how these are formed. Constructivism describes how an individual consolidates their own knowledge through the discussion of and sharing of knowledge with others in the same “field” (75). When students enter an animal or veterinary science degree, they begin constructing their own academic identity (76), which can be greatly influenced by the practitioners they observe. So where might animal welfare education be compromised by a hidden curriculum? The most obvious place is when animals used in teaching are used without consideration for their welfare, prioritising attainment above the animals, e.g., where live animals are used when simulations could do, or clear “end points” not being used to dictate where an animal is no longer to be used in demonstrations. There has been some research in these areas already, for example the use of cadavers in US high schools for dissections was discussed by Suiter et al. (77) who highlighted that a clear dissection policy will include discussion with the class about their preferred method. They also stated that students should not receive a penalty for choosing not to participate.

More work exploring what types of hidden curricula develop in animal science could help ensure animal welfare light on the potential welfare challenges.

#### Animal-Centred Education

Patient-centred care highlights the patient as an individual, and pushes the care of that individual away from what is easy for the medical practitioners, what is easy for automated systems to measure, or what is easy for hospitals to record, and instead highlights the importance of the individual's experience in their medical journey (78). The concept is relevant for animal welfare education not because patient-centred care is necessarily a fully developed model with a clear definition, but because of the ethos behind it. It may also help to tackle some of the concerns previously mentioned about hidden curriculums. The emphasis on the individual should be an innately comfortable stomping ground for the animal welfare scientist, as animal welfare is often defined as the individual's experience as it copes with its environment.

If animal science degrees adopt an “animal-centered” education approach, what could we expect to change? This work is already being explored in some zoos, with a recognition that some animals are more tolerant of being “on-exhibit” (79, 80) than others, and changing zoo management in response to these individual needs. This might work in a variety of ways. Glick and Moore (81) discussed whether ongoing digital revolution could lead to patient-centred education in the medical curriculum, through using technology to support relationships between clinician and patient. They discussed how networked care systems allow clinicians to have an understanding of the patient's history prior to consultation. It is common in animal courses, from animal care to zoology, to have “teaching animals.” With a little investment, a database could be implemented which logs the interactions each animal has in teaching, and allows for considered use over time. Students could be encouraged to contribute to and manage such a database (teaching valuable information skills as a bonus), such as deciding what information is relevant to include. Each year, as part of general feedback on the course, students could be asked to review their thoughts on the animals included in the course, or some volunteers give a “handover” round to incoming students.

Many of the approaches to animal-centred education have additional benefits, perhaps forming more holistic communities of practice [see (82)]. Given the prevalence of untreated mental illness in the UK student body (83), these approaches could be incorporated into general well-being reform.

#### Blueprinting Assessment

Assessment “blueprinting” refers to the overt linking of learning outcomes (or instructional objectives) to assessment, e.g., fully describing what someone should be able to do after an educational intervention. Learning outcomes have a common stem, a description of the behaviour, and an assessable outcome (24). For example, after reading this paper, the reader will be able to list some gaps in the animal welfare education literature. We could assess this by asking the reader to list materials, and award points for correct identification.

Assessment is a hot topic within higher education research. Assessment and feedback are integrally linked, and one cannot exist without the other. Both are powerful influencers on student behaviour (84), and feedback is often the more tricky element, often conceptualised as a commodity passed from marker to student (85). The basic principle is that we use multiple criteria to judge a student's piece of work, and not all of these are entirely objective [indeed, within disciplines we often see co-constructed understandings of the discipline as a sort of “academic literacy” which is not always legible to those outside of a discipline, see (86)]. Therefore, students require multiple opportunities to practice these skills and receive feedback on them in order to improve (87).

As a case study, Patil et al. (88) reported on a workshop they had run to expose their pathology teaching staff to the concept of blueprinting. The faculty staff gathered to scope and define the purpose of assessment in their course, decide weightings, and discuss the available methods. Their students reported satisfaction with the new assessments, and staff considered the changes meaningful. There are a number of exercises that are open source and which incorporate blueprinting assessment into programme or course design such as the CAIeRO^1^ process and the ELDeR process^2^. The ELDeR process was core to the development of the new Agricultural BScs at Edinburgh (89) and sparked rigorous discussion about how and why assessments run as they do.

Blueprinting assessments can be particularly relevant for animal welfare course because of the aforementioned reliance on practical and critical reasoning skills. When a piece of animal welfare work is interdisciplinary, as many student theses are, it can become extremely pertinent to give students the opportunity for feedback on these skills at earlier stages. Imagine a short animal welfare course with a single assessment, an essay discussing the importance of the human-animal bond. A blueprinting approach would identify what learning outcomes that essay would cover, and then be able to identify at what point students would receive an opportunity within the course to practice the skill and receive relevant feedback prior to submission of the essay. This approach by nature encourages more assessment, although not necessarily more summative assessment. The role of formative assessment in animal welfare has been poorly explored, but is likely to improve the student's ability to parse assessment criteria (90), and therefore improve their academic literacy within an interdisciplinary topic such as animal welfare science.

#### Authentic Assessment

Related to the idea of blueprinting assessments is that of authentic assessment. It is now fashionable in higher education to talk of assessment as learning, which promotes the student's role in their own learning process (64). Authentic assessment is one attempt to address this by returning the context of practice to an assessment. Unsurprisingly, there are varying conceptualisations of authentic assessment in the literature. Gulikers et al. (91) provided a framework for authentic assessment encompassing: task; physical context; social context; assessment form; and assessment criteria. Many animal welfare assessments incorporate some level of authentic assessment, e.g., encouraging the collection of behavioural data and incorporating this into the write-up of a report. This would cover elements of task, physical context (being in the animal's environment), as well as aspects of form and criteria (grading a product that they will likely have to understand or reproduce in practice). There is already some discussion in the literature regarding authenticity of assessment, particularly social context, such as in Zuin et al. (56).

Role play is used in animal welfare teaching to simulate the social context (44) students will find themselves in. As educators have reported on the perceived tension between different ethical standpoints in animal welfare teaching (50, 55), this may also be a valuable tool for teaching. Authentic assessments could incorporate recognition of “wicked” problems, challenges which are considered complex, open-ended and intractable (92). Wicked problems are widely discussed in terms of agriculture (93), public policy (92), and economics (94). The assessment of such involves an understanding of not only how physiology and psychology relate to animal welfare, but also how networks should be managed in an attempt to encourage multiple partners to move toward a goal. In essence, how can a group form a flexible and innovative enough network in order to achieve the aims of a wicked challenge (95)? One component of a grade is that it ought to inform potential employers in some manner about how the student should be expected to perform in a relevant task (96), and so assessments which also grade elements that we value when we tackle wicked problems (e.g., teamwork, etc.) as well as the knowledge and critical analysis the majority of higher education assessments focus on.

An authentic assessment for animal welfare science could have a strong focus on social context to recognise the communicative nature of the roles these student scientists are likely to take on. However, it should also incorporate more forms and criteria. Given the vast prevalence of animal-related content online, students could create an educational resource for the public. This is a product which many will need to create in their own future employment, and reflects a range of skills that are challenging to assess in a short-form essay.

We also know what challenges face animal science students in their likely career paths. Shelter workers experience euthanasia related strain (97), and feel unprepared for work environments with limited resources and upskilling potential owners (98). An authentic assessment might capture some form of this in criteria, such as asking students to justify what resources they prioritise. A task-orientated authentic assessment might ask students to work in an environment where euthanasia occurs, if there is convincing evidence that many animal science graduates may need to work with this task.

There are challenges with authentic assessment, such as accountability and cost (99), however there are numerous descriptions of case studies incorporating authentic assessment in a range of disciplines (100–102). For animal welfare science educators aiming to explore authentic assessment the answer is likely not wholesale change but exploring what graduates currently feel unprepared for to produce an assessment that will help them learn how to *practice* their discipline, not simply assess knowledge.

## Discussion

Perceived gaps in animal welfare teaching are not a novel finding, with the earliest paper included in this literature search being a letter in the Journal of the American Veterinary Medical Association calling for humane education (103). The importance of embedding this education within animal-related curricula has been a recurring topic of discussion over the last few decades (35, 48, 57, 104, 105). However, this review has found that this call is rarely framed in terms of pedagogical design. This is not a criticism, indeed animal welfare science can be said to be a relatively new science, and one which has had to prove its credentials, both as a scientific discipline (106), but also to recognise the importance of animal sentience (107). In other, perhaps more established fields, discipline based educational research is still relatively new. For example, the journal Chemistry Education: Research and Practice began in 2000 (108). We can benefit from the extensive experience in medical education however, as the Journal of Veterinary Medical Education recently published a 50 year retrospective (109), and in the first issue of Medical Teacher, one article advised the use of an overhead projector as an incoming technology (110). Human education and behavioural change for animal welfare is increasingly becoming a topic of interest, with the foundation of the Human Behavioural Change for Animal Welfare conference in 2016 (111) and animal welfare is a common topic in the annual Veterinary Education conference run by the Veterinary Schools Council, with international reach (112). The purpose of this review was both to highlight some key areas where animal welfare science could explore from an educational research standpoint, and to make educational research more accessible to those within the animal welfare discipline. There has been an increase in the number of publications, likely reflecting the increase in scholarly output that has been observed over the past decade (113), and the challenge for DBER is to parse this literature alongside the literature of their own specialism. Fraser's (12) contention is that specialists who teach must have an understanding of educational research too, at least enough to critically appraise their own practice. This is particularly challenging in STEMM educational research where the interplay between the humanities-born discipline of educational research and the STEMM fields can be difficult for the STEMM researcher to work with [see discussion in (114)]. In this review, I aimed to purposefully select gaps which had been lightly explored within the literature, or had particular relevance to animal welfare research, to provide budding animal welfare DBER practioners with a basis to explore further. I hope I have demystified some of the terminology and provided an accessible introduction to concepts within DBER, but this will only prove to be true should the field develop its own DBER practices. These may very well be practices that I have not conceived of in this review.

There are other aspects of educational research which can and should be explored within animal welfare. A theory-led approach, perhaps focussing on cultural perspectives, individualism, action theory, or positivist vs. constructionist epistemological stances may offer new perspectives on how we teach animal welfare. These approaches may be more suited to fields like veterinary education where the field of work is more developed, as in the body of work in Perrin (72), however there are more students of animal welfare than in the veterinary field, and our research ought to include those too.

## Conclusions

Animal welfare should incorporate education research in its interdisciplinary approach. There are a number of gaps in the current literature base which could be explored within animal welfare science education, such as hidden curricula, blueprinting assessment, animal-centred education, and authentic assessment. These approaches are particularly missing outwith veterinary education research, and could offer new perspectives on how we teach animal welfare across a variety of courses.

## Author Contributions

JM conceived of the study, performed all analyses, and wrote the manuscript in its entirety.

### Conflict of Interest

The author declares that the research was conducted in the absence of any commercial or financial relationships that could be construed as a potential conflict of interest.

## Footnotes

^1^*Introduction to CAIeRO*. ILT—The University of Northampton. Available online at: https://www.northampton.ac.uk/ilt/academic-development/caiero/ (accessed June 13, 2018).

^2^*University of Edinburgh About ELDeR*. The University of Edinburgh. Available online at: https://www.ed.ac.uk/information-services/learning-technology/supporting-learning-and-teaching/learning-design/about (accessed June 29, 2018).
